# Comprehensive genomics, systems, and structural assessment for novel target identification in penicillin-resistant *Streptococcus pneumoniae*

**DOI:** 10.3389/fbinf.2026.1784287

**Published:** 2026-04-29

**Authors:** Avani Panickar, Suvitha Anbarasu, Anand Manoharan, Sudha Ramaiah, Anand Anbarasu

**Affiliations:** 1 Medical and Biological Computing Laboratory, School of Biosciences and Technology, Vellore Institute of Technology (VIT), Vellore, Tamil Nadu, India; 2 Department of Biosciences, School of Biosciences and Technology, Vellore Institute of Technology (VIT), Vellore, Tamil Nadu, India; 3 Department of Biotechnology, School of Biosciences and Technology, Vellore Institute of Technology (VIT), Vellore, Tamil Nadu, India; 4 Infectious Diseases Medical and Scientific Affairs, GlaxoSmithKline (GSK), Worli, Maharashtra, India

**Keywords:** alternative target, antimicrobial resistance, essential gene analysis, genome-wide association study, membrane-protein simulation, oligopeptide permease

## Abstract

**Introduction:**

The increasing prevalence of penicillin-resistant *Streptococcus pneumoniae* (PRSP) has compromised the efficacy of conventional β-lactam therapies, and the inefficiency of penicillin-binding proteins (PBPs) as reliable drug targets further underscores the urgent need to explore novel alternatives. The current study employs an *in silico* strategy that integrates genomics, genome-wide association studies (GWASs), network analyses, and membrane protein simulations to systematically identify and prioritize new antimicrobial targets.

**Methodology:**

A total of 665 PRSP genomes from Indian clinical isolates collected between 1996 and 2022 were analyzed. High-quality genome assemblies were annotated and used for pangenome construction and GWASs to identify gene clusters associated with penicillin resistance. Candidate genes were further prioritized through essentiality screening, functional annotation, subcellular localization prediction, evolutionary conservation analysis, druggability assessment, and structural modeling.

**Results:**

Integrated analysis identified OppC2, an essential oligopeptide permease of the ABC transporter family, as a highly favorable drug target. Network and functional enrichment analyses linked OppC2 to transport-associated pathways relevant to pneumococcal survival and adaptation. Structural modeling revealed a high-confidence protein model with a druggable binding pocket, while molecular dynamics simulations confirmed the stability of the structure in a physiological membrane environment.

**Conclusion:**

This comprehensive approach enabled the identification of conserved, essential, and accessible drug targets within PRSP populations, providing an adaptable framework to guide next-generation antimicrobial target identification beyond traditional PBPs.

## 1 Introduction

*Streptococcus pneumoniae* (*S. pneumoniae*) is a leading cause of life-threatening infections, including pneumonia, meningitis, and sepsis, accounting for over 1.2 million deaths globally each year. The burden is disproportionately high in children and older adults, who are particularly vulnerable to these infections due to weakened immune function, in the latter population sometimes resulting from age-related processes (Cilloniz et al., 2024). The problem is particularly severe in India, where *S. pneumoniae* contributes to approximately 23% of the global pneumonia burden and causes over 100,000 deaths annually among children under five. The case fatality rate for invasive pneumococcal disease is estimated at 17.8% in adults and reaches nearly 34.9% in elderly populations (Jayaraman et al., 2019; Nagaraj et al., 2025). Despite the availability of antibiotics and pneumococcal conjugate vaccines, increasing resistance to penicillin remains a major concern as penicillin has long been a first-line treatment for pneumococcal infections. Recent surveillance studies in India have reported that penicillin non-susceptibility rates exceed 30% among clinical isolates, attributable to factors including the widespread overuse of antibiotics and the pathogen’s capacity to undergo genetic recombination and horizontal gene transfer (Torumkuney et al., 2025). Our previous studies on Indian pneumococcal genomes further validated these resistance patterns, demonstrating that penicillin-binding proteins (PBPs), the primary targets of penicillin antibiotics, are frequently mutated in resistant strains, thereby highlighting the molecular basis of penicillin resistance (Panickar et al., 2025b; Panickar et al., 2025a).

Traditional methods for target identification have generally involved laboratory-based screening approaches that focus on essential genes, often failing to capture the complex, polygenic nature of antibiotic resistance observed in clinical settings (Mobegi et al., 2017). Genome-wide association studies (GWASs) in bacterial pathogens have emerged as powerful tools for identifying loci associated with penicillin response while accounting for population structure and phylogenetic relationships, thereby overcoming the limitations of traditional methods (Earle et al., 2016). The shortcomings of single-gene-targeting strategies have become increasingly evident as bacteria exhibit remarkable adaptability through network-based resistance mechanisms involving multiple interconnected pathways rather than isolated genetic factors (Diagne et al., 2022). This adaptability is further reflected in the emergence of multidrug-resistant phenotypes that cannot be explained by mutations in individual target genes but instead arise from coordinated changes across functional modules, including efflux pumps, stress response systems, and metabolic pathways, which collectively enable bacterial survival under antibiotic pressure (Miryala et al., 2019).

Computational genomics approaches based on whole-genome sequences of *S. pneumoniae* have recently been applied to identify potential therapeutic targets (Gohain et al., 2025). However, currently available drugs for the treatment of *S. pneumoniae* infections are increasingly compromised by the emergence of drug-resistant strains. Therefore, the identification of new therapeutic targets is crucial. To address the increasing threat of penicillin-resistant *S. pneumoniae* (PRSP), this study aimed to identify novel therapeutic targets with favorable characteristics for antimicrobial development, providing a framework for resistance-focused drug discovery in the genomics era. Our study integrates comparative genomics, GWASs, network interaction analyses, and molecular dynamics simulations to identify conserved, essential, and druggable targets beyond traditional PBPs, offering a strategic foundation for the development of novel therapeutics targeting PRSP.

## 2 Materials and methods

### 2.1 Genome collection and quality assessment

A total of 665 *S. pneumoniae* genomes were retrieved from the Global Pneumococcal Sequencing (GPS) database, focusing on Indian isolates with associated penicillin-susceptibility data. Genome assembly quality was assessed using QUAST v5.2, a widely used tool for evaluating assembly metrics (Gurevich et al., 2013). Genome completeness was further evaluated using BUSCO v6.0, which performs benchmarking based on evolutionarily conserved single-copy orthologs to ensure reliable genome representation (Manni et al., 2021).

### 2.2 Genome annotation and pangenome analysis

Genome annotation was performed using Prokka v1.14.6 for gene prediction and structural annotation, including coding sequences (CDSs) and repeat regions (Seemann, 2014). For pangenome analysis, annotated GFF files generated by Prokka were used as input for both Panaroo v1.3.3 and Roary v3.13.0. Panaroo was used to generate the core genome alignment (Tonkin-Hill et al., 2020), whereas Roary was employed to obtain gene presence–absence matrices, summarize pangenome distribution, and generate outputs used for phylogenetic analysis based on the core genome (Page et al., 2015).

### 2.3 Resistance phenotyping and genome-wide association study

Minimum inhibitory concentration (MIC) values were retrieved from the database and converted into binary phenotypes for susceptibility analysis. Isolates classified as susceptible were assigned a value of 0, while those classified as intermediate or resistant were grouped together and assigned a value of 1. GWASs were performed using Pyseer v1.3.6 (Lees et al., 2018). The input for this analysis was the gene presence–absence matrix obtained from the pangenome analysis. To control population structure and clonal relatedness among isolates, Pyseer was run using a linear mixed model (LMM) framework. A relatedness matrix derived from the core genome SNP alignment was incorporated into the model as a random effect, allowing correction for lineage effects and reducing false-positive associations caused by shared ancestry. This approach enabled the identification of genetic variants significantly associated with penicillin resistance while accounting for population structure.

### 2.4 Protein–protein interaction network analysis

To prioritize candidate genes identified from GWAS, a protein–protein interaction (PPI) network analysis was performed to identify highly connected hub proteins that may play central roles in resistance-associated pathways. Interaction networks for the significant genes were constructed using the STRING (Search Tool for the Retrieval of Interacting Genes/Proteins) v12. 0 database (Szklarczyk et al., 2023), which integrates both physical and functional associations derived from experimental data, curated databases, and computational predictions. A medium-confidence score threshold (0.4–0.6) was applied to retain reliable interactions for downstream analysis (Priyamvada et al., 2025). The resulting networks were imported into Cytoscape v3.10.1 for visualization and analysis (Shannon et al., 2003). Key hub genes within the networks were identified using the cytoHubba plugin v0.1, which ranks nodes based on multiple topological parameters. The analysis included degree, maximum neighborhood component (MNC), maximal clique centrality (MCC), closeness, betweenness, and stress centrality metrics to identify highly connected nodes (Chin et al., 2014). First-level interaction clusters of the prioritized hub genes were extracted from the network for further investigation. Functional pathway enrichment analysis for the significant genes and their interactors was performed using the Gene Ontology (GO) and Kyoto Encyclopedia of Genes and Genomes (KEGG) annotations integrated within the STRING platform to identify biological pathways associated with PRSP (Kanehisa, 2000).

### 2.5 Functional annotation and localization prediction

To characterize the biological roles of the prioritized hub genes, functional annotation was performed using eggNOG-mapper v2.1.9, based on the eggNOG database v5.0 of orthologous groups (Cantalapiedra et al., 2021). This tool enables accurate functional transfer across sequences using ortholog-based inference. The annotation results included predicted protein functions, KEGG pathway assignments, GO terms, and Clusters of Orthologous Groups (COG) classifications to identify the functional categories associated with the candidate genes (Popov et al., 2025). To further validate the predicted functional domains of selected genes, InterProScan v5.63 and the Pfam database v36.0 were used for domain identification. InterProScan integrates multiple protein signature databases to predict conserved domains and functional motifs, while Pfam provides curated protein family models based on hidden Markov models (HMMs) (Mistry et al., 2021; Paysan-Lafosse et al., 2023). The subcellular localization of the selected proteins was predicted using a multi-tool approach to improve prediction reliability. PSORTb v3.0 was used as the primary localization predictor because of its high accuracy for bacterial proteins. Additional predictions were obtained using CELLO v2.5, DeepLoc v2.0, and BUSCA v1.0, providing complementary validation of localization results (Yu et al., 2010; Thumuluri et al., 2022).

### 2.6 Essentiality and human homology filtering

To identify essential genes among the GWAS-significant loci, essentiality was evaluated using the Database of Essential Genes (DEG v15.2), a curated repository of experimentally validated essential genes across multiple organisms (Luo et al., 2021). Protein sequences of the prioritized candidate genes were compared against the DEG database to assess their similarity to known essential genes. To ensure that the selected genes represent pathogen-specific antimicrobial targets, homology screening was performed against the human proteome. Protein sequences of the selected targets were analyzed using BLASTp v2.13.0 against the *Homo sapiens* reference protein database of the National Center for Biotechnology Information (NCBI). Genes showing significant similarity to human proteins (e-value <0.01 and sequence identity >30%) were carefully evaluated to minimize potential host cross-reactivity, with preference given to genes showing minimal similarity to human proteins (Altschul, 1997).

### 2.7 Structure prediction and pocket mapping

In the absence of an experimentally determined three-dimensional structure for the selected candidate protein, structural modeling was performed using AlphaFold v2.3, an AI-based algorithm that predicts protein structures directly from amino acid sequences with high accuracy (Satkanov and Chupakhin, 2024). AlphaFold employs deep learning techniques to generate initial protein folds and iteratively refine atomic-level structures by incorporating evolutionary, physical, and geometric constraints. The quality of the predicted protein structure was validated using multiple complementary approaches. Initial validation was conducted using the SAVES v6.0 meta-server, which integrates multiple validation tools to assess overall structural quality (Lüthy et al., 1992). Ramachandran plot analysis was performed, and structures with >90% of residues in favored regions were considered acceptable for further analysis. Additionally, ProSA-web v2 was used to evaluate the overall quality of the predicted structure by comparing it against experimentally determined structures in the Protein Data Bank through Z-score calculations (Wiederstein and Sippl, 2007). For binding-site prediction, DoGSiteScorer v1.0 was used. This automated web server combines geometric and grid-based algorithms to detect potential binding pockets by mapping the protein structure onto a Cartesian grid and identifying cavities based on geometric criteria. Binding pockets were ranked and characterized based on multiple descriptors, including volume, surface area, and druggability scores. The highest-scoring pocket was selected for further analysis based on optimal size, accessibility, and drug-like properties (Volkamer et al., 2012).

### 2.8 Molecular dynamics simulation

Molecular dynamics (MD) simulations were carried out using GROMACS software v2024.2 to evaluate the intrinsic stability of the modeled protein. Simulations in both aqueous and membrane environments were conducted to reflect physiologically relevant conditions consistent with the predicted subcellular localization of the target protein (Peela et al., 2023).

#### 2.8.1 Lipid-bilayer environment simulations

For membrane-associated simulations, the target protein was embedded in a dipalmitoylphosphatidylcholine (DPPC) bilayer using the CHARMM-GUI Membrane Builder (Wu et al., 2014). The system topology was prepared using the CHARMM36 force field with Berger lipid parameters (Berger et al., 1997). Energy minimization was performed for 50,000 steps using the steepest descent algorithm with a convergence criterion of 1,000 kJ mol^−1^ nm^−1^ (Joshi et al., 2024). The system underwent NVT equilibration for 100 ps using the Nosé–Hoover thermostat, followed by NPT equilibration for 100 ps with semi-isotropic pressure coupling using the Parrinello–Rahman barostat to allow membrane relaxation. Long-range electrostatic interactions were handled using the particle mesh Ewald (PME) method with a real-space cutoff of 1.0 nm and Fourier spacing of 0.16 nm. Following equilibration, three independent 300 ns production MD simulations were performed to evaluate the stability and reproducibility of the protein structure in a lipid-bilayer environment. The resulting trajectories were analyzed to assess structural stability, conformational flexibility, and protein–environment interactions (Peela et al., 2023).

#### 2.8.2 Aqueous environment simulations

MD simulations were also conducted in an aqueous environment using the CHARMM36 force field (Huang et al., 2017). Each protein structure was centered in a dodecahedral simulation box and solvated with TIP3P water molecules under periodic boundary conditions, maintaining a minimum distance of 1.0 nm between the protein and box edges. Energy minimization was performed using the steepest descent algorithm for 50,000 steps, with a convergence tolerance of 1,000 kJ mol^−1^ nm^−1^. System equilibration was conducted in two phases to achieve physiological conditions (310 K). First, NVT (canonical ensemble) equilibration was performed for 100 ps using the V-rescale thermostat to stabilize temperature. This was followed by NPT (isothermal–isobaric) equilibration for 100 ps using the Parrinello–Rahman barostat for pressure coupling (Parrinello and Rahman, 1981). Long-range electrostatic interactions were calculated using the PME method with a real-space cutoff of 1.2 nm, PME order of 4, and a Fourier spacing of 0.16 nm (George et al., 2025). Following equilibration, three independent 300 ns production MD simulations were performed in the aqueous environment, and the resulting trajectories were used for structural stability and conformational analyses.

## 3 Results

### 3.1 Genome analysis

#### 3.1.1 Genome dataset and quality statistics

**FIGURE 1:**
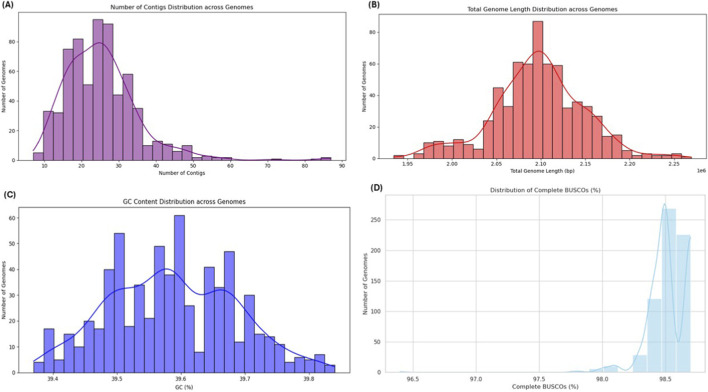
Quality assessment metrics of the 665 *S. pneumoniae* genomes. **(A)** Distribution of contig counts across genomes, **(B)** Total genome length distribution, **(C)** GC content distribution across genomes, and **(D)** Distribution of complete BUSCO percentages.

A total of 665 *S. pneumoniae* genome sequences were retrieved from the GPS database, focusing on isolates from India with penicillin MIC values ranging from ≤0.03 μg/mL to 4 μg/mL (Supplementary Table S1). Genome assembly quality ranged from 7 contigs to 87 contigs, with an average of 24.6 contigs (Figure 1A). Total genome length varied between 1,934,410 bp and 2,269,304 bp, consistent with the expected genome size of *S. pneumoniae* (Figure 1B). GC content showed minimal variation across isolates, ranging from 39.37% to 39.84%, with an average of 39.55%, consistent with the known GC content range of *S. pneumoniae* (Figure 1C; Supplementary Table S2). Genome completeness assessed using BUSCO indicated high-quality assemblies, with most genomes showing near-complete gene sets suitable for downstream genomic analysis (Figure 1D; Supplementary Table S3).

#### 3.1.2 Gene prediction and pangenome structure

**FIGURE 2:**
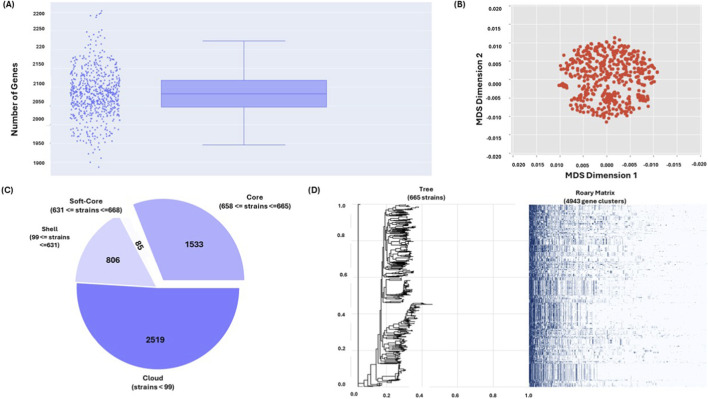
Genome quality assessment and pangenome analysis of 665 *S. pneumoniae* isolates: **(A)** Panaroo QC summary showing assembly statistics and annotation consistency across genomes, **(B)** Outlier detection and sample-level quality metrics, **(C)** Pangenome breakdown into core, soft-core, shell, and cloud clusters genes, and **(D)** Core-genome phylogeny.

Genome annotation revealed consistent gene content across the analyzed genomes. The average number of predicted genes per genome was 2,214, ranging from 1,995 to 2,440 genes. The majority were coding sequences (CDSs), with an average of 2,082 CDSs per genome (range: 1,887–2,303). Non-coding RNA elements were also identified. On average, each genome contained 80 miscellaneous RNAs (miscRNAs), 5 ribosomal RNAs (rRNAs), 47 transfer RNAs (tRNAs), and 1 transfer–messenger RNA (tmRNA), consistent with typical pneumococcal genomes (Supplementary Table S4). Pangenome analysis highlighted substantial genetic diversity within the studied *S. pneumoniae* population. The total pangenome comprised 4,943 genes. Among these, 1,533 genes were classified as core genes shared across all genomes, representing conserved essential functions. The accessory genome included 85 soft-core genes, 806 shell genes, and 2,519 cloud genes, reflecting varying levels of gene prevalence across isolates (Figures 2A–D).

#### 3.1.3 Resistance traits and GWAS findings

Phenotypic analysis revealed the distribution of penicillin susceptibility and non-susceptibility among the 665 *S. pneumoniae* isolates. Of these, 384 isolates were classified as susceptible, while 281 were classified as intermediate or resistant to penicillin. This phenotype distribution formed the basis for subsequent GWAS analysis. Following quality control, 4,943 variants were retained for association testing across all isolates. Bonferroni correction was applied for multiple testing, yielding a genome-wide significance threshold of p ≤ 1.01 × 10^−5^. The GWAS identified 11 genetic variants associated with penicillin resistance, of which a subset exceeded the Bonferroni-corrected genome-wide significance threshold, while others showed suggestive associations, with effect sizes ranging from −0.163 to 0.159 with *p*-values ranging from 2.76 × 10^−6^ to 1.07 × 10^−4^. The estimated narrow-sense heritability (h^2^) was 0.15, indicating that approximately 15% of the phenotypic variance in penicillin resistance could be attributed to genetic factors (Supplementary Figure S1; Table 1). The association plot showed the distribution of −log_10_ (*p*-values) for all tested variants, with the horizontal line representing the Bonferroni-adjusted genome-wide significance threshold. Most variants remained below the threshold, while a limited number of loci exceeded it, indicating significant associations with penicillin resistance. The quantile–quantile (QQ) plot compared the observed and expected *p*-value distributions. Most variants followed the expected null distribution, indicating minimal inflation of association statistics. The deviation observed at the upper tail corresponded to significant loci and suggested that the linear mixed model implemented in Pyseer effectively controlled for population structure while preserving true association signals. This moderate heritability suggests that while genetic factors contribute to resistance, additional mechanisms may influence antibiotic susceptibility in pneumococcal populations. Interestingly, significant loci displayed a mix of positive and negative effect sizes, suggesting that pneumococcal genomes contain both resistance-promoting and susceptibility-modulating genetic components.

**TABLE 1:** Significant genomic loci associated with penicillin-susceptibility profiles identified across the 665 *S. pneumoniae* genomes.

Variant	Allele frequency	Filter-*p*-value	lrt-*p*-value	Beta	Beta-std error	Variant_h^2^	Antibiotic
nanM	0.217	0.00465	2.76E-06	−0.163	0.0345	0.181	PEN
gsiC	0.203	0.0311	6.47E-06	−0.152	0.0333	0.174	PEN
oppC_2	0.203	0.0311	6.47E-06	−0.152	0.0333	0.174	PEN
oppC_1	0.203	0.0311	6.47E-06	−0.152	0.0333	0.174	PEN
mngA	0.391	0.614	0.000107	0.159	0.0409	0.15	PEN
tkt_1	0.391	0.614	0.000107	0.159	0.0409	0.15	PEN
tkt_2	0.391	0.614	0.000107	0.159	0.0409	0.15	PEN
fruA_2	0.391	0.614	0.000107	0.159	0.0409	0.15	PEN
manP	0.391	0.614	0.000107	0.159	0.0409	0.15	PEN
manR	0.391	0.614	0.000107	0.159	0.0409	0.15	PEN
alsE	0.391	0.614	0.000107	0.159	0.0409	0.15	PEN

### 3.2 Systems analysis

#### 3.2.1 Protein interaction network analysis

**FIGURE 3:**
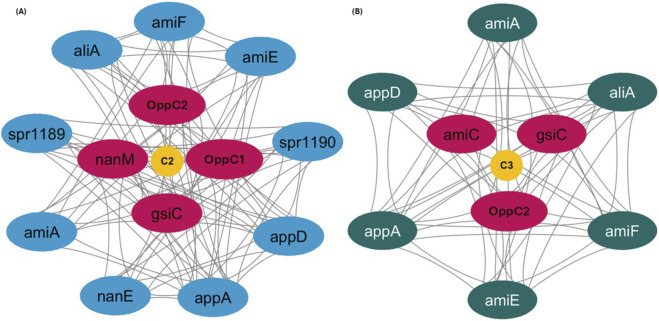
Key functional clusters identified in the interaction network: **(A)** Cluster C2, highlighting genes associated with transport and metabolism, forming a tightly connected functional module. **(B)** Cluster C3, showing a second highly interconnected module enriched for OppC2-centered interactions involving related transport proteins. Node colors represent cluster groups.

To prioritize candidate genes identified through GWAS, PPI network analysis was performed to detect highly connected hub proteins that may play central roles in resistance-associated pathways. A total of 11 significant genes were identified from the GWAS analysis, of which 8 were successfully mapped to the interaction network. These proteins formed a network comprising 17 nodes and 129 interactions, with confidence scores ranging from 0.4 to 0.6 (Supplementary Figure S2). The interaction network was visualized using the cytoHubba plugin to rank genes based on network topology, and the top 5 hub genes were selected for each scoring parameter (Supplementary Figure S3). From this analysis, three genes (*mngA*, *oppC2*, and *gsiC*) were identified as key interactors. Network clustering partitioned the interaction network into three overlapping clusters (C1, C2, and C3), with 17 unique genes represented across these clusters. Cluster C1, centered on *mngA*, contained 4 nodes and 8 edges, representing the smallest cluster. Cluster C2, focused on *gsiC*, comprised 13 nodes with 104 edges, including 4 of the test genes. Cluster C3, centered on *oppC2*, consisted of 9 nodes with 62 edges and included 3 of the test genes (Table 2). Given the smaller node size and lower connectivity of cluster C1 compared with the other two clusters, C1 was excluded from further analysis, and subsequent investigations focused on C2 and C3 (Figures 3A,B). The hub genes identified through this network analysis were subsequently subjected to functional characterization and essentiality filtering to identify potential antimicrobial targets.

**TABLE 2:** Summary of PPI clusters formed by penicillin response-associated genes.

Cluster no	Cluster source node	No. of test proteins in the cluster	No. of nodes	No. of edges
C1	GsiC	3	4	8
C2	OppC2	4	13	104
C3	MngA	3	9	62

#### 3.2.2 Functional categories and localization

**FIGURE 4:**
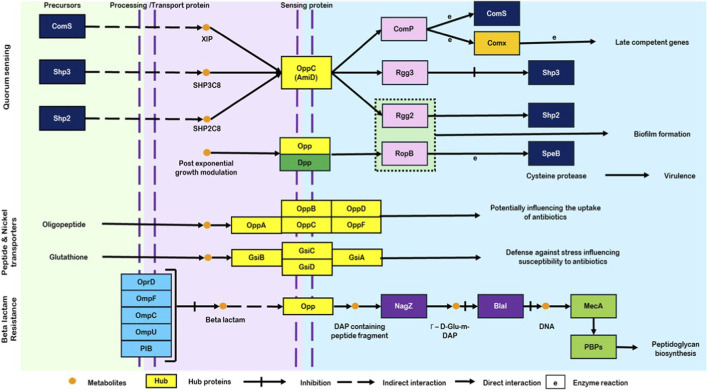
Overview of quorum sensing, peptide transport, and β-lactam resistance pathways in *S. pneumoniae*.

Functional enrichment analysis revealed distinct GO profiles among the cluster genes. Cluster C2 demonstrated significant enrichment in biological processes related to peptide transport, protein transport, and transmembrane transport. Enriched cellular components indicated localization to the membrane-associated transporter complexes and the plasma membrane, consistent with ATP-binding cassette (ABC) transporter systems. Molecular function analysis revealed predominant peptide transmembrane transporter activity. Cluster C3 exhibited similar GO enrichment patterns across biological processes and cellular components to those observed in C2. However, molecular function analysis revealed additional enrichment in broader transmembrane transporter activity beyond the peptide-specific transport observed in C2. The combined test genes encompassed all identified GO terms and also showed additional enrichment in organic substance transport under biological processes. COG categories, and KEGG pathway analyses identified key functional pathways associated with both clusters (Figure 4). The major enriched pathways found in clusters C2 and C3, and the test genes included ABC transporters, β-lactam resistance mechanisms, and quorum-sensing pathways (Table 3). Specific COG classifications revealed that *OppC2* belongs to category U and functions as a permease protein (OppC2), whereas *gsiC* is classified under category P as an inner membrane component of a binding protein-dependent transport system (Supplementary Figure S4). This localization analysis consistently supports their classification as membrane-associated transport proteins, further validating the functional assignments of OppC2 and GsiC within binding protein–dependent transport systems (Table 4).

**TABLE 3:** Summary of enriched GO terms and KEGG pathways for interacting proteins.

Category	Term ID	Term description	Observed gene count	Background gene count	Strength	False discovery rate	Interacting proteins
GO process	GO:0015833	Peptide transport	8	12	1.93	2.61E-10	aliA, appD, appA, amiF, amiE, amiD, amiC, amiA
GO process	GO:0015031	Protein transport	6	18	1.63	4.20E-06	aliA, appA, amiE, amiD, amiC, amiA
GO process	GO:0071702	Organic substance transport	9	137	0.92	3.53E-05	aliA, fruA, appD, appA, amiF, amiE, amiD, amiC, amiA
GO process	GO:0055085	Transmembrane transport	11	258	0.74	4.51E-05	aliA, fruA, appD, appC, appB, appA, amiF, amiE, amiD, amiC, amiA
GO function	GO:1904680	Peptide transmembrane transporter activity	3	7	1.74	0.04	aliA, appA, amiA
GO component	GO:0005886	Plasma membrane	9	260	0.65	0.0057	aliA, fruA, appC, appB, appA, amiE, amiD, amiC, amiA
GO component	GO:0030288	Outer membrane-bounded periplasmic space	3	8	1.68	0.0057	aliA, appA, amiA
GO component	GO:0043190	ATP-binding cassette (ABC) transporter complex	3	30	1.11	0.0305	aliA, appA, amiA
KEGG	spr02024	Quorum sensing	10	52	1.39	1.84E-10	aliA, appD, appC, appB, appA, amiF, amiE, amiD, amiC, amiA
KEGG	spr01501	Beta-lactam resistance	5	12	1.73	3.80E-06	amiF, amiE, amiD, amiC, amiA
KEGG	spr02010	ABC transporters	5	78	0.91	0.0088	amiF, amiE, amiD, amiC, amiA

**TABLE 4:** Subcellular localization predictions for the protein based on multiple databases.

Tools	Localization prediction	Scores	
		GsiC	OppC2
CELLO prediction	Cytoplasmic membrane	4.885	4.778
	Extracellular region	0.034	0.1
	Cytoplasm	0.057	0.082
	Cell wall	0.024	0.04
PSORTb	Cytoplasmic membrane	10	10
	Cell wall	0	0
	Cytoplasm	0	0
	Extracellular region	0	0
DeepLoc	Cytoplasmic membrane	0.2371	0.9999
	Extracellular region	0.0836	0.0001
	Cell wall and surface	0.2438	0

#### 3.2.3 Essentiality and host homology filtering

Essentiality evaluation revealed that *gsiC* demonstrated 32% identity with essential genes in the database, whereas *OppC2* showed 100% identity to essential genes, exceeding the minimum threshold of 30% identity used for essential gene classification. Given this threshold, *OppC2* was selected for further analysis because of its complete identity match and was subsequently used for structural modeling and molecular dynamics simulations. Pangenome presence–absence analysis further showed that the OppC transporter family was present across all analyzed *S. pneumoniae* isolates, indicating that this permease belongs to the conserved core genome. To ensure that the selected target would not exhibit cross-reactivity with human proteins, homology screening was performed against the *H. sapiens* protein database using BLAST analysis. No significant sequence similarity was observed between OppC2 and human proteins, confirming the absence of potential off-target effects. This lack of homology supports the suitability of OppC2 as a selective antimicrobial target that would minimize adverse effects on host cells (Table 5).

**TABLE 5:** Similarity results for the proteins against essential gene databases.

Query ID	gsiC	OppC2
Subject ID	DEG10070047	DEG10070104
Pct identity	30.357	100
Alignment length	56	308
No. of mismatches	38	0
Gap openings	1	0
Q start	84	1
Q end	138	308
S start	296	1
S end	351	308
e-value	1.6	0
Bit score	23.5	618

### 3.3 Structural biology analysis

#### 3.3.1 Structural model and binding sites

**FIGURE 5:**
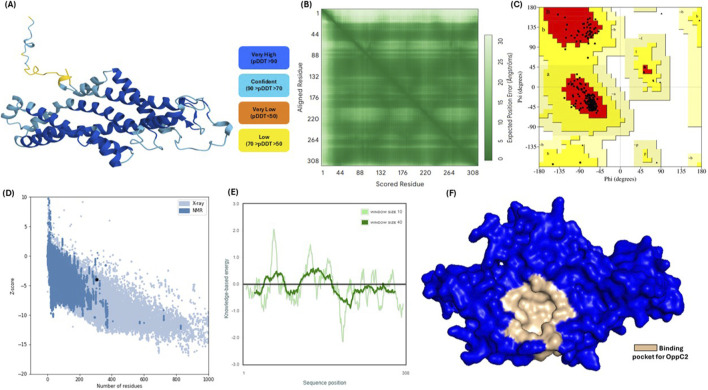
Structural modeling and validation of the predicted OppC2 protein structure. **(A)** Predicted three-dimensional structure of OppC2, colored according to predicted Local Distance Difference Test confidence scores from N- to C-terminus. **(B)** AlphaFold predicted aligned error (PAE) heatmap showing residue-wise confidence in the relative positioning of residues within the model. **(C)** Ramachandran plot illustrating the distribution of backbone dihedral angles (φ and ψ), indicating the stereochemical quality of the model. **(D)** ProSA-web Z-score plot comparing the predicted OppC2 structure with experimentally determined protein structures, **(E)** sliding-window energy profile showing local model quality across the protein sequence, **(F)** predicted binding pocket of OppC2 (the solvent-accessible surface of the protein is shown in blue, with the putative ligand-binding pocket highlighted in beige, indicating a well-defined and accessible cavity potentially involved in substrate or inhibitor binding).

Due to the absence of an experimentally validated three-dimensional structure for the OppC2 protein, structural modeling was performed using AlphaFold. The predicted model adopted a well-defined α-helical structure compatible with the expected fold of the protein. The calculated confidence scores showed that most regions of the protein exhibited high confidence (>90%, depicted in dark blue), with some regions showing medium confidence (>70%, light blue) and a minority showing low confidence (>50%, yellow). Notably, no region showed very low confidence scores, indicating overall structural reliability. The predicted template modeling (pTM) score, which measures the accuracy of the overall structure, was 0.84. This value exceeds the commonly used threshold of 0.5 for reliable global topology prediction, indicating high confidence in the overall fold of the protein (Figure 5A). Predicted aligned error (PAE) analysis further confirmed that most regions exhibited low positional uncertainty, indicating reliable residue-level accuracy (Figure 5B). Ramachandran plot analysis demonstrated excellent stereochemical quality, with 95.7% of residues located in the most favored regions and 4.3% in additionally allowed regions. Importantly, no disallowed conformations were observed, further confirming the structural reliability of the model (Figure 5C). Quality validation using ProSA yielded a Z-score of −3.93, which falls within the range typically observed for experimentally determined protein structures of similar size (Figure 5D). The energy profile showed consistently negative values across the sequence, with no significant indications of structural irregularities or steric clashes (Figure 5E).

To evaluate the therapeutic potential of OppC2, binding-site analysis was conducted using DoGSiteScorer. The predicted binding pocket exhibited favorable characteristics for drug targeting, with a volume of 497.04 Å^3^, a surface area of 663.43 Å^2^, and a depth of 17.94 Å (Figure 5F). The binding pocket was located within a chemically diverse, and therefore adaptable, environment comprising hydrophobic residues (LEU, ILE, PHE, and VAL), polar residues (SER, THR, and GLN), and aromatic residues (TRP and TYR). The analysis identified 32 hydrophobic interaction sites and 130 total pocket atoms, with 48 hydrogen-bond acceptors and 6 donors available for potential ligand interactions. These analyses yielded a druggability score of 0.79, suggesting that the OppC2 binding pocket is highly amenable to small-molecule modulation, further supporting its potential as a viable therapeutic target (Table 6).

**TABLE 6:** Structural and physicochemical characteristics of the predicted binding pockets, including residue-level composition and druggability metrics of OppC2.

Parameter	Value	Essential AA	Presence in the pocket
Hydrophobic interactions	32	ALA	1
Volume	497.04	ARG	0
Enclosure	0.15	ASN	0
Surface	663.43	ASP	0
Depth	17.94	CYS	0
Surf/Vol	1.33476179	GLN	1
Ell c/a	0.13	GLU	0
Ell b/a	0.65	GLY	1
Site Atms	130	HIS	0
Accept	48	ILE	5
Donor	6	LEU	1
Hydrophobicity	0.37	LYS	0
Cs	93	MET	3
Ns	13	PHE	2
Os	22	PRO	2
Ss	2	SER	3
Polar AA	0.37	THR	3
Apolar AA	0.63	TRP	1
Simple score	0.31	TYR	2
Drug score	0.790831	VAL	2

#### 3.3.2 Molecular dynamics outcomes

##### 3.3.2.1 Lipid bilayer environment

**FIGURE 6:**
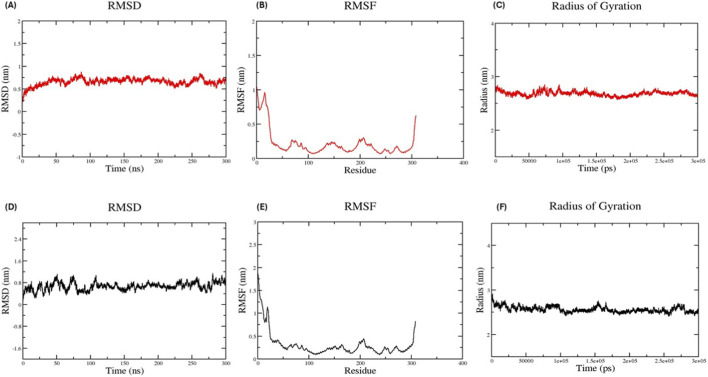
MD simulation analysis of OppC2 during 300 ns timeframe: **(A)** RMSD profile of OppC2 during membrane simulation in a DPPC bilayer (red), **(B)** RMSF showing residue-wise fluctuations of OppC2 in the membrane environment (red), **(C)** Rg indicating structural compactness during the membrane simulation (red), **(D)** RMSD profile of OppC2 during aqueous simulation (black), **(E)** RMSF showing residue-wise fluctuations in the aqueous environment (black), and **(F)** Rg indicating structural compactness during the aqueous simulation (black).

To understand the structural behavior of OppC2 under physiologically relevant conditions, three independent 300 ns MD simulations were performed with the protein embedded in a DPPC bilayer. The root mean square deviation (RMSD) profiles showed an average deviation of 0.88 ± 0.24 nm, indicating that the trajectories reached a stable conformational regime following the initial equilibration phase and maintained overall structural stability throughout the simulation (Figure 6A). The root mean square fluctuation (RMSF) revealed an average fluctuation of 0.28 ± 0.06 nm, suggesting limited residue mobility across most regions of the protein, particularly within the transmembrane helices, while higher flexibility was primarily confined to terminal and loop regions (Figure 6B). The radius of gyration (Rg) remained stable with an average value of 2.63 ± 0.05 nm, indicating preservation of the overall compact structural organization of OppC2 within the lipid environment (Figure 6C). Hydrogen-bond analysis showed an average of 430.17 ± 2.15 bonds, reflecting stable intraprotein interactions and reduced water-mediated contacts due to partial embedding within the hydrophobic membrane core (Supplementary Figure S5A). Similarly, the solvent-accessible surface area (SASA) averaged 195.22 ± 2.72 nm^2^, indicating stable solvent accessibility in the membrane environment (Supplementary Figure S5B). The potential energy trajectory remained stable throughout the simulation, averaging approximately −2.15 × 10^6^ kJ/mol, confirming the structural stability of OppC2 in the membrane environment (Supplementary Figure S5C).

##### 3.3.2.2 Aqueous environment simulations

For comparison, OppC2 was also simulated in an aqueous environment using the same triplicate 300 ns simulations. The RMSD profile showed an average deviation of 0.74 ± 0.06 nm, indicating that the protein retained structural stability in solution (Figure 6D). RMSF values averaged 0.34 ± 0.03 nm, suggesting that residue fluctuations were mainly restricted to terminal and loop regions, while the core structural domains remained stable (Figure 6E). The Rg remained stable at 2.54 ± 0.02 nm, confirming preservation of the overall compact protein conformation (Figure 6F). Hydrogen-bond, SASA, and potential energy analyses showed average values of 522.35 ± 2.60 bonds, 183.43 ± 2.16 nm^2^, and −3.06 × 10^6^ kJ/mol, respectively, reflecting greater solvent exposure and increased water-mediated interactions compared with the membrane environment (Supplementary Figure S5D–F). Overall, OppC2 maintained structural integrity in both environments. However, the membrane simulations provide a more physiologically relevant representation of protein behavior, consistent with its predicted role as a membrane-associated transporter (Table 7).

**TABLE 7:** Summary of structural and dynamic parameters for the protein under aqueous and lipid bilayer simulation environments.

Simulation	Aqueous	Lipid bilayer
Average RMSD (nm)	0.94	0.62
Average RMSF (nm)	0.38	0.22
Average Rg (nm)	2.5	2.6
Average SASA (nm^2^)	188.87	191.91
Average PE (kJ/mol)	−3062609	−2155241
Average Hydrogen Bond	520.33	428.01

## 4 Discussion

The escalating crisis of antibiotic resistance among *S. pneumoniae* strains poses a major challenge in clinical management, particularly in India. Recent surveillance data indicate that only 41.2% of isolates remain susceptible to penicillin under standard breakpoints, implying non-susceptibility rates approaching 60%. However, susceptibility increases substantially when high-dose breakpoints are applied (Nagaraj et al., 2025). These findings underscore the urgent need for innovative strategies to identify alternative targets. This study provides a comprehensive genomic framework integrating GWAS, systems biology, and structural modeling to identify novel therapeutic targets against penicillin-resistant pneumococcal infection. The quality assessment demonstrated that the 665 pneumococcal genomes exhibited consistent metrics, including an average contig count of 24.6, GC content of 39.6%, and 98.5% BUSCO completeness, which closely align with established pneumococcal genomic characteristics reported in landmark studies (Croucher et al., 2011; Chewapreecha et al., 2014). Annotation analysis revealed approximately 2,214 genes per genome, closely matching the average of ∼2,200 genes reported in comprehensive pneumococcal genome analyses, thereby validating our dataset (Donati et al., 2010). The pangenome comprised 4,943 genes, including 31% core genes and 69% accessory genes, highlighting the remarkable genetic flexibility that supports pneumococcal adaptation under antibiotic pressure. This observation is consistent with previous pangenome studies showing that accessory genes constitute the majority of pneumococcal genetic diversity (Andam and Hanage, 2015). The distribution of phenotypic traits, with 42.3% penicillin non-susceptibility, indicates a substantial clinical burden in the population and supports subsequent GWAS analyses. We identified 11 significantly associated genes with a moderate heritability estimate, challenging the traditional view of resistance as a simple monogenic trait. These findings are consistent with recent bacterial GWASs demonstrating that complex traits often exhibit polygenic structures, where multiple loci contribute small but cumulative effects (Lehtinen et al., 2017; Mobegi et al., 2017). The moderate heritability observed further suggests that environmental factors and epistatic interactions also play important roles in determining resistance phenotypes. PPI network analysis revealed functionally consistent clustering of genes associated with penicillin response, with ABC transporters acting as central hubs. This finding is consistent with previously reported experimental evidence highlighting the critical role of ABC transporters in pneumococcal virulence and antibiotic resistance (Garmory and Titball, 2004; Basavanna et al., 2009).

The identification of *OppC2*, *mngA*, and *gsiC* as key hub genes directly aligns with signature-tagged mutagenesis studies that identified multiple ABC transporters as essential for pneumococcal pathogenesis in animal models (Basavanna et al., 2009). Specifically, the LivJHMGF branched-chain amino acid transporter identified by Basavanna et al. (2009) showed similar network centrality to our hub genes, further supporting the biological relevance of our predictions. Functional enrichment analysis revealed the predominant involvement of these genes in peptide transport, protein transport, and transmembrane transport activities, consistent with experimental studies characterizing pneumococcal ABC transporters. The GO profiles identified closely match functional annotations from biochemical studies of the Ami-AliA/B oligopeptide permease system, which plays roles in both nutrient acquisition and the regulation of virulence factors (Kerr et al., 2004). The clustering of OppC2 and gsiC within transport-related pathways further supports experimental evidence that oligopeptide transporters regulate multiple virulence genes and contribute to pneumococcal pathogenesis through both direct nutritional effects and indirect regulatory mechanisms (Wang et al., 2005). Among the hub genes identified in the interaction network, OppC2 was selected for detailed structural and functional analysis based on multiple complementary criteria, including its association with penicillin response in GWAS, its central position within transport-related pathways, predicted membrane localization, and essentiality. Essentiality analysis confirmed that OppC2 shows 100% identity with experimentally validated essential genes, providing strong support from experimental datasets across multiple bacterial species. In addition, the conservation of the OppC transporter family across pneumococcal genomes further points to its potential as a broadly relevant target. Signature-tagged mutagenesis in Group A *Streptococci* has shown that disruption of OppA, the substrate-binding component of the same operon containing OppC2, resulted in a significant reduction in virulence in animal infection models, including reduced mortality and tissue damage (Zheng et al., 2018). Similar findings in *Vibrio alginolyticus* demonstrated that suppression of the *opp* genes led to deficiencies in adhesion, biofilm formation, hemolytic activity, and overall virulence, further supporting the essential role of this transport system across bacterial pathogens (Liu W. et al., 2017). Importantly, the biological role of OppC2 within the oligopeptide permease system provides insight into how this transporter may influence β-lactam resistance phenotypes. OppC2 forms the membrane permease component of the OppABC transporter complex, which mediates ATP-dependent uptake of extracellular oligopeptides rather than antibiotic efflux. Consequently, OppC2 is unlikely to function as a classical drug efflux pump. Instead, its contribution to antibiotic response is likely indirect and mediated through physiological pathways associated with peptide transport. Oligopeptide permeases are known to regulate bacterial metabolism by supplying amino acids required for cell wall biosynthesis and by participating in peptide-based signaling pathways involved in virulence and stress adaptation (Maio et al., 2016). In *S. pneumoniae,* peptide transport systems have also been linked to regulatory networks controlling virulence factor expression and environmental adaptation. As β-lactam antibiotics disrupt peptidoglycan synthesis, alterations in peptide uptake and nutrient availability may influence cell wall homeostasis, thereby affecting susceptibility to these antibiotics (Garai et al., 2017). The identification of OppC2 as an essential membrane protein in this study, therefore, suggests that it may function as a physiological modulator of β-lactam response rather than a direct resistance determinant, consistent with studies demonstrating that ABC transport systems broadly contribute to pneumococcal survival and pathogenicity (Borezee et al., 2000).

In AlphaFold-based structural modeling, exceptional quality metrics were obtained, including a pTM score of 0.84% and the finding that 95.7% of residues were in Ramachandran-favored regions, exceeding commonly accepted benchmarks for drug discovery applications. Recent validation studies have demonstrated that AlphaFold models with pTM scores greater than 0.8 provide reliable foundations for structure-based drug design, particularly for membrane proteins that have historically been challenging targets (Borkakoti and Thornton, 2023; Yang et al., 2023). The successful modeling of OppC2 demonstrates that AI-based structural prediction can help overcome traditional limitations in membrane protein crystallography, thereby facilitating structural investigation of challenging membrane proteins. This achievement is particularly significant given that ABC transporters represent one of the largest membrane protein families and have been extensively validated as antimicrobial targets in experimental studies. Binding-pocket analysis identified a highly druggable site (druggability score 0.79) with favorable geometric and chemical properties, consistent with studies reporting successful targeting of bacterial ABC transporters, including inhibitors against the PatA/PatB system in multidrug-resistant pneumococci (Garvey et al., 2011). The chemical diversity of the pocket, incorporating hydrophobic, polar, and aromatic residues, mirrors the binding-site characteristics of known druggable ABC transporters and suggests good potential for optimization. The identified binding pocket is derived from structural modeling and druggability prediction and therefore represents a predicted ligand-binding region rather than direct experimental confirmation. Nevertheless, the consistency between AlphaFold structural prediction, pocket characterization, and membrane molecular dynamics simulations provides strong structural support for the presence of a stable and potentially druggable site. Such computational structural analyses are widely used in antimicrobial discovery to identify potential targets prior to experimental validation (Bianchi et al., 2012). Experimental studies have shown that ABC transporter inhibitors can restore antibiotic sensitivity in resistant bacteria, supporting the therapeutic potential of the selected target (Amarnani et al., 2023). MD simulations further validated the biological relevance of the structural model, showing enhanced stability in lipid-bilayer environments. These findings are consistent with reported experimental studies of ABC transporter function, which demonstrate that membrane embedding is essential for proper folding and conformational transitions of the protein (How et al., 2025). The reduced flexibility observed in the transmembrane regions during membrane simulations aligns with biophysical studies, indicating that the lipid bilayer stabilizes membrane protein dynamics while still permitting essential conformational changes required for transport. Hydrogen-bonding patterns and structural compactness were maintained throughout the simulations, supporting the conclusion that this model accurately represents a native-like protein conformation (Varghese et al., 2022).

The results have important implications for understanding pneumococcal resistance mechanisms and for the development of targeted therapeutic strategies. OppC2 was identified as an essential, membrane-localized transporter whose presence is associated with penicillin response, suggesting that it modulates physiological pathways influencing β-lactam response rather than acting as a classical resistance determinant. This finding addresses a key limitation in traditional drug discovery, where targets identified *in vitro* often fail to translate into clinical efficacy. The oligopeptide permease system plays a dual role in nutrient acquisition while also contributing to bacterial pathogenicity. The moderate heritability observed in our resistance analysis suggests that OppC2-targeted interventions may be more effective in combination therapies, given that OppC2 is enriched among susceptible isolates and appears to support the physiological state that favors β-lactam responsiveness. This observation aligns with emerging clinical evidence indicating that combination therapies are more effective against β-lactam-resistant pneumococci and are less prone to resistance evolution than single-agent treatments (Cremers et al., 2019). Recent studies of bacterial ABC transporter inhibitors have also demonstrated synergistic effects when combined with conventional antibiotics, supporting the potential for OppC2-targeted strategies as β-lactam sensitizers in combination treatments (How et al., 2025). Although the present study provides strong computational evidence supporting OppC2 as a potential antimicrobial target, experimental validation is necessary to confirm its functional role in pneumococcal physiology and antibiotic response. Future studies could utilize CRISPR interference (CRISPRi) or targeted gene deletion approaches to evaluate the effects of OppC2 perturbation on bacterial viability and β-lactam susceptibility. CRISPRi systems have recently emerged as powerful tools for functional genomics and essential gene analysis in bacterial pathogens, including *S. pneumoniae* (Liu X. et al., 2017; Jana et al., 2024). In addition, gene complementation assays, growth and MIC measurements, and transcriptomic profiling under antibiotic stress conditions could further clarify the physiological role of OppC2 in peptide transport and cell envelope homeostasis. For membrane transporters such as OppC2, substrate uptake or peptide transport assays may provide additional insights into how disruption of this system influences bacterial metabolism and antibiotic susceptibility. Such studies would provide critical biological validation of OppC2 function and strengthen its potential as a therapeutic target (Lin et al., 2025; Boudza et al., 2026). Importantly, the high-quality structural model we have presented provides a foundation for virtual screening, while the identified interaction networks suggest opportunities for combination therapies targeting multiple resistance pathways simultaneously. This study demonstrates that modern computational approaches can accelerate antimicrobial discovery while maintaining clinical relevance. It provides a framework for addressing the ongoing antibiotic resistance crisis through innovative target-identification strategies.

## 5 Conclusion

Analysis of PRSP genomes indicates that resistance arises from complex interactions among genetic, structural, and functional adaptations rather than from single-gene mutations. By integrating population genomics, GWAS, protein–protein interaction networks, and structural bioinformatics, we identified OppC2, an essential oligopeptide permease, as a potential drug target. The identification of its essential role in bacterial survival, absence of human homologs, and a chemically diverse, druggable binding pocket highlights the effectiveness of computational approaches in antimicrobial discovery. High-confidence AlphaFold modeling, supported by MD simulations, further demonstrates the therapeutic relevance of OppC2 and the potential of such approaches to accelerate drug design for challenging targets such as membrane proteins. Overall, this study provides a systematic framework for the identification of novel therapeutic targets against PRSP infections, emphasizing the value of integrative computational strategies in addressing emerging antibiotic resistance.

## Data Availability

The datasets presented in this study can be found in online repositories. The names of the repository/repositories and accession number(s) can be found in the article/Supplementary Material.
