# Supplementary material for: Comprehensive genomics, systems, and structural assessment for novel target identification in penicillin-resistant Streptococcus pneumoniae
Source: Front Bioinform. 2026 Apr 29;6:1784287. doi: 10.3389/fbinf.2026.1784287 (PMC13168064; doi:10.3389/fbinf.2026.1784287)
Supplement: Supplementary file 3 [file Supplementaryfile1.docx]

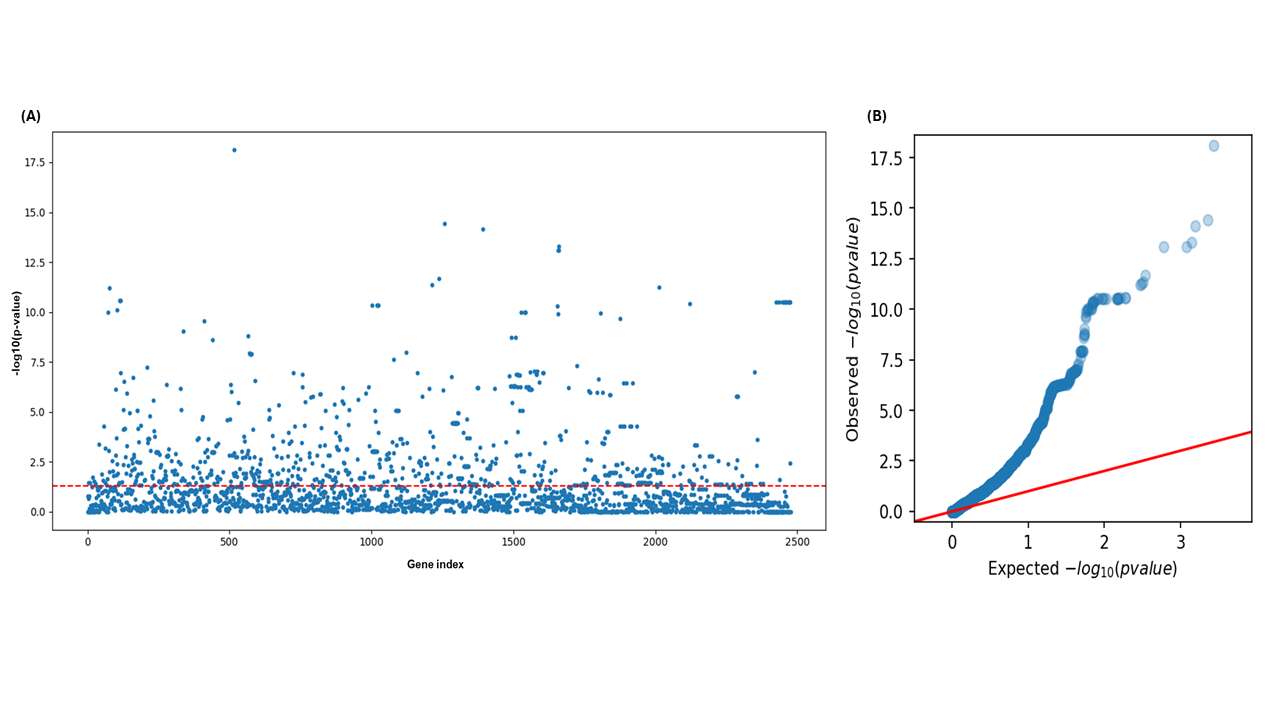


**Supplementary Figure S1:** Gene presence–absence GWAS results for penicillin resistance in 665 *S. pneumoniae* genomes **(A)** Manhattan-style plot showing –log10(p-values) for all gene clusters tested. The red dashed line marks the significance threshold. **(B)** Q–Q plot comparing observed versus expected –log10(p-values).


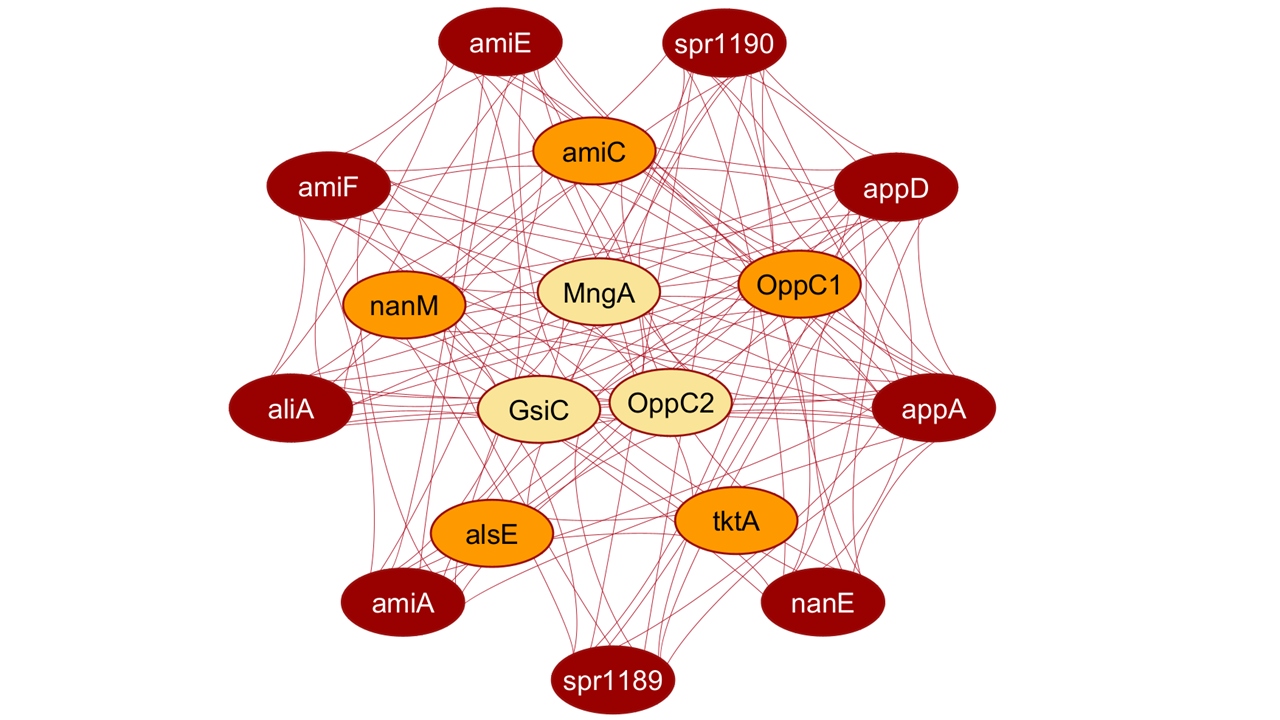


**Supplementary Figure S2.** Protein–protein interaction network connectivity**.** Red nodes represent the overall interaction network, yellow nodes indicate GWAS-identified genes, and the central nodes such as OppC2, GsiC, and MngA show extensive interactions.


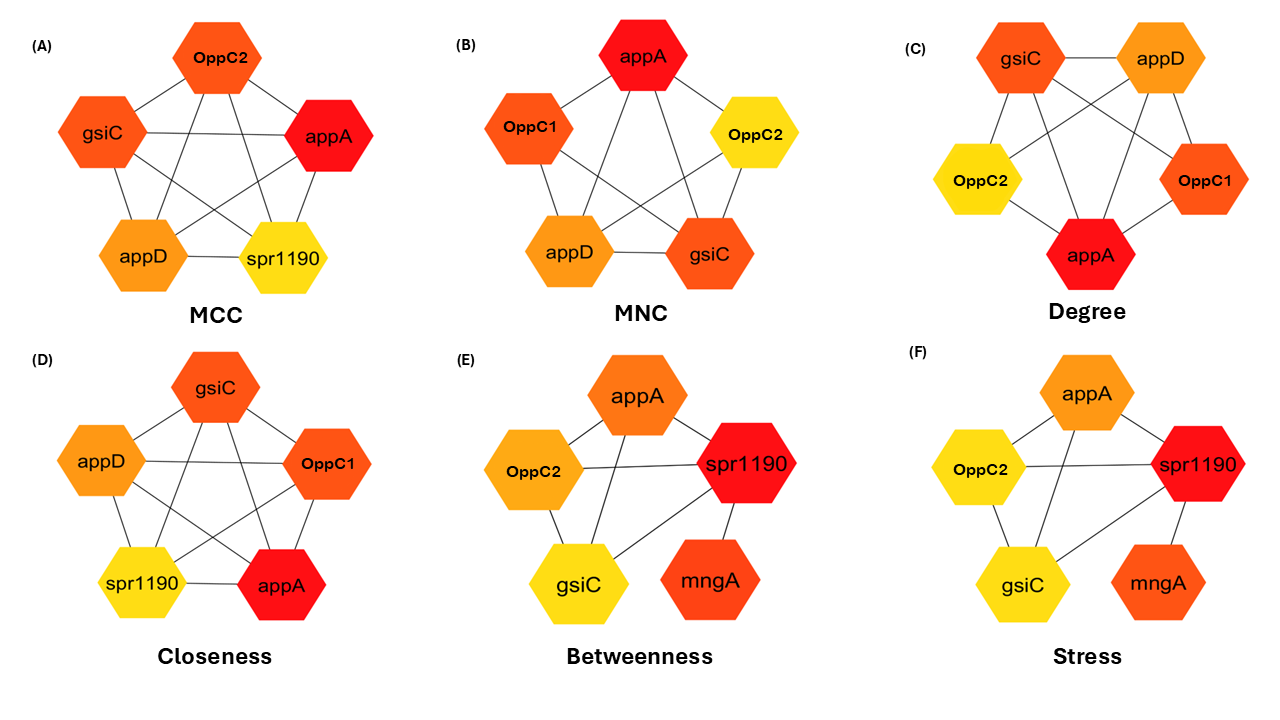


**Supplementary Figure S3:** Hub gene identification **(A)** MCC, **(B)** MNC, **(C)** Degree, **(D)** Closeness, **(E)** Betweenness, and **(F)** Stress centrality rankings of candidate genes within the interaction network. Each panel shows the top-scoring nodes, with color intensity reflecting higher centrality scores.


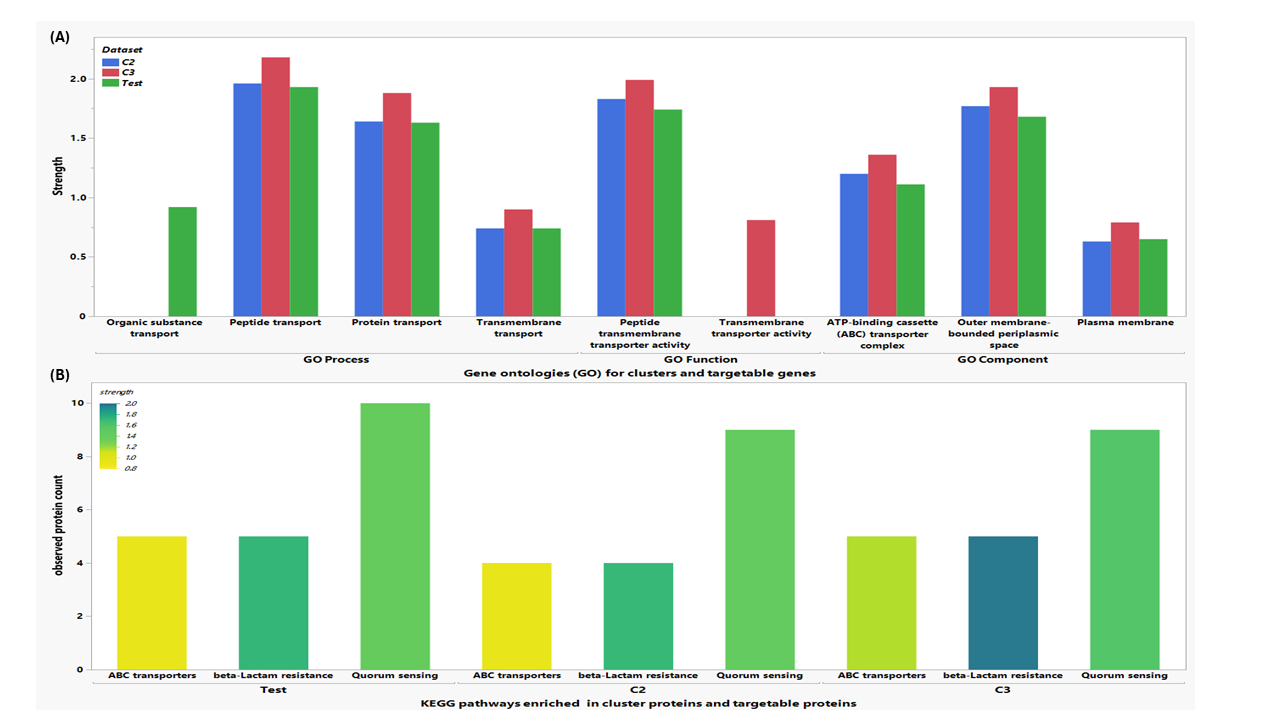


**Supplementary Figure S4:** Functional enrichment of cluster genes and targetable proteins **(A)** GO term enrichment (process, function, and component) for clusters C2, C3, and targetable proteins**(B)** KEGG pathway enrichment showing involvement of ABC transporters, β-lactam resistance pathways, and quorum sensing across cluster and targetable gene sets.


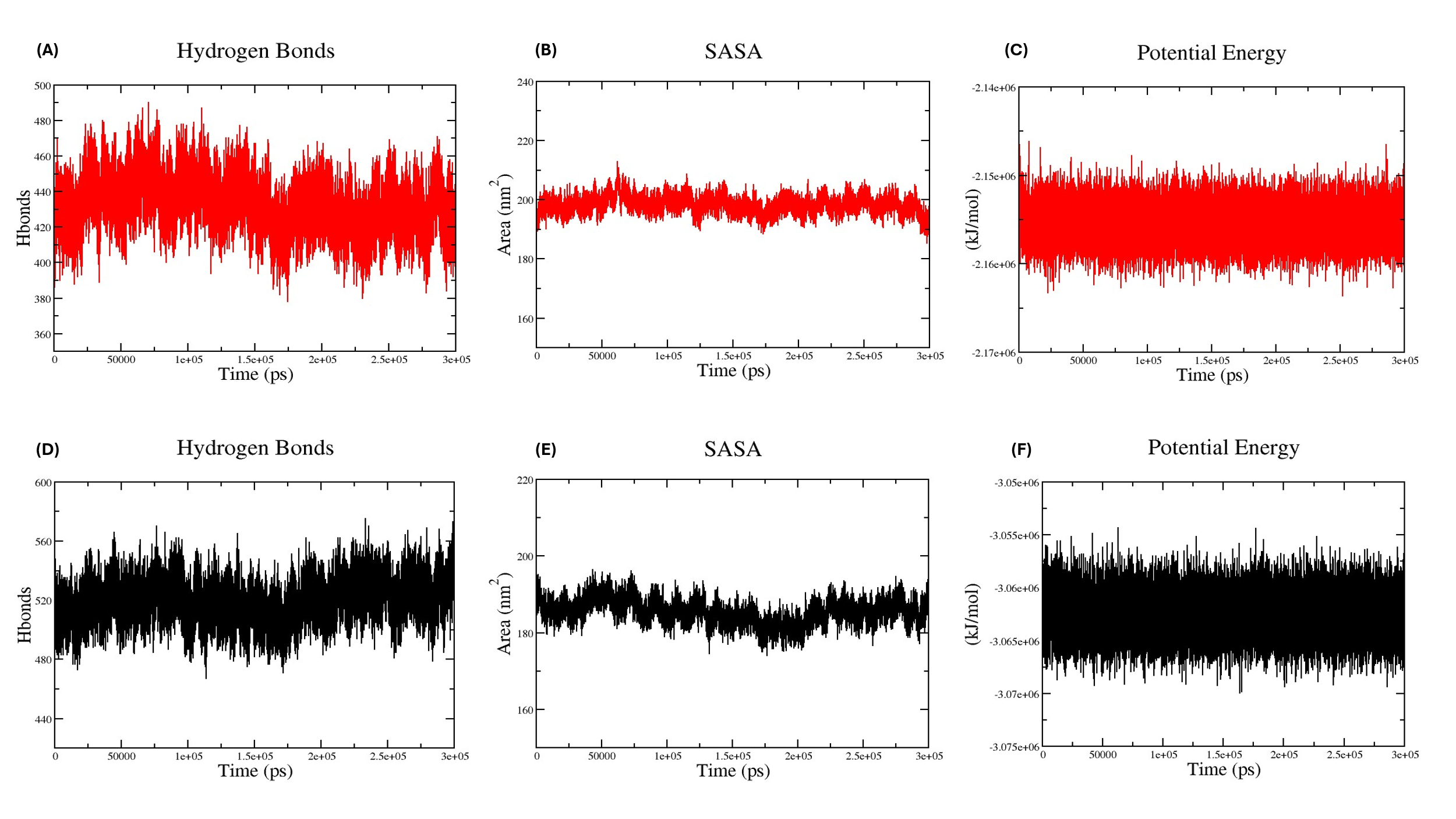
**Supplementary Figure S5:** MD simulation analysis of OppC2 during 300 ns timeframe: **(A)** Hydrogen bond profile during membrane simulation (DPPC bilayer) shown in red, **(B)** SASA during membrane simulation shown in red, **(C)** Potential energy profile during membrane simulation shown in red, **(D)** Hydrogen bond profile during aqueous simulation shown in black, **(E)** SASA during aqueous simulation shown in black, **(F)** Potential energy profile during aqueous simulation shown in black, allowing comparison of OppC2 structural stability under membrane and aqueous environments.
